# Healthcare professionals’ perceptions of implementing a decision support intervention for cascade screening for beta-thalassemia in Pakistan

**DOI:** 10.1038/s41431-022-01074-1

**Published:** 2022-03-01

**Authors:** Shenaz Ahmed, Hussain Jafri, Wajeeha Naseer Ahmed, Muhammed Faran, Yasmin Rashid, Yasmin Ehsan, Mushtaq Ahmed

**Affiliations:** 1 Leeds Institute of Health Sciences, University of Leeds, Leeds, UK; 2 Thalassaemia Society Pakistan, Fatima Jinnah Medical University, Lahore, Pakistan; 3 Thalassaemia Society Pakistan, Lahore, Pakistan; 4 Health Department Punjab, Lahore, Pakistan; 5 Yorkshire Regional Genetics Service, Leeds, UK

**Keywords:** Genetic counselling, Medical ethics

## Abstract

Counselling relatives of individuals with βeta-Thalassaemia Major (β-TM) about cascade screening is the role of field officers (FOs) in the Punjab Thalassaemia Prevention Project (PTPP). This paper presents FOs’ views about using a ‘decision support intervention for relatives’ (DeSIRe) to facilitate informed decision making, and their perception of its implementation and sustainability. Semi-structured qualitative interviews were conducted with nine FOs (June to July 2021) in seven cities in the Punjab province (Lahore, Sheikhupura, Nankana Sahab, Kasur, Gujranwala, Multan, and Faisalabad) following its use in routine clinical practice. Thematic analysis shows that the FOs were overwhelmingly supportive of the DeSIRe, expressing enthusiasm and identifying benefits of its use, both for their own practice and for relatives. They supported the aim of the intervention to enable them to facilitate relatives’ decision-making about cascade screening, and advocated its use more widely within the PTPP and the other provinces of Pakistan. Overall, the DeSIRe was valued by the FOs for use in routine practice. These findings suggest the DeSIRe is highly likely to be implemented by healthcare professionals more widely in the PTPP and, therefore, is worth evaluating to prove its efficacy. Further research is needed on the extent to which the DeSIRe could be adapted for use by other healthcare professionals with similar responsibilities in the other provinces of Pakistan, and in other low-middle income countries.

## Introduction

βeta-Thalassaemia Major (β-TM) is the most prevalent genetic disorder in Pakistan. Estimated figures suggest there are approximately 100,000 individuals with β-TM in Pakistan, and this figure continues to increase annually by 7000–9000 children born with the condition [1, 2]. The treatment required by individuals with β-TM, mainly blood transfusions and chelation therapy, places psychological and financial stress on families, and a burden on Pakistan’s healthcare system [1]. The Punjab Thalassaemia Prevention Project (PTPP) is a government-funded intervention which aims to reduce the incidence of β-TM in the Punjab province of Pakistan.

The PTPP has nine regional centres covering 36 districts in the Punjab province, with over 100 professionals, including 48 male Field Officers (FOs). A key strategic approach to reducing the incidence of β-TM includes the offer of cascade screening. This targeted screening is crucial in a country with limited healthcare facilities and resources, where consanguineous marriages are the norm and genetic literacy is low [3]. In the PTPP, FOs approach parents of children with β-TM in thalassaemia clinics to obtain their consent to approach their relatives. Working with consenting parents, FOs organise family meetings with relatives at their homes to provide information about the condition and offer carrier testing. On average, these family meetings are attended by approximately twenty family members, ranging in age, gender, relationship to the child, and literacy levels. However, the PTPP has observed that the uptake of cascade screening is suboptimal.

There is little research on the reasons for the low uptake of carrier screening by relatives, although a contributing factor may be the lack of resources specifically for FOs, to enable FOs to facilitate relatives’ decision-making. Therefore, we developed a decision support intervention for relatives (DeSIRe) of children with β-TM. The paper-based DeSIRe was developed in Urdu for use by FOs in face-to-face consultations to facilitate informed decisions about cascade screening. Details of how the DeSIRe was developed can be found elsewhere [4].

The DeSIRe was based on the principle of shared decision-making (SDM), a patient-centred process in which healthcare professionals work with individuals to reach a decision about their healthcare [5]. The use of decision support interventions can support SDM [6]. Decision support interventions are evidence-based tools that usually include information about the health condition, and highlight the benefits, risks, probabilities, and uncertainties of the different options, enabling individuals to consider their values, based on their own preferences, and to make an informed decision [7]. The use of decision support interventions in clinical practice can improve communication between healthcare professionals and patients, improve patient knowledge and their understanding of the decision-making process, reduce decisional conflict, and improve patients’ satisfaction with their decisions [8].

Various factors can influence whether or not healthcare professionals adopt a decision support intervention. Factors that can enable adoption include the provision of training on how to use the intervention, clarification of the significance of SDM, and how the intervention could have a positive impact on patients’ decision-making [9]. Factors that can hinder adoption include healthcare professionals’ perceptions about its effectiveness, concerns about the time and patience that would be required to explain the information in routine clinical practice, and concerns about how it may be perceived by potential users (patients or their relatives) [9]. However, there is sparse research on healthcare professionals’ views in Pakistan about the enablers and barriers to adopting a decision support intervention.

Overall, the concepts of SDM and decision support interventions are novel in a low-middle income country such as Pakistan. Also, the adoption of a decision support intervention in clinical practice would require changes in work culture, so an implementation strategy for such an intervention should be based on healthcare professionals’ perceptions of the enablers and barriers to its use. Therefore, this study aimed to explore FOs’ experiences and views of using the DeSIRe in routine clinical practice, and their perceptions of its implementation and sustainability in the PTPP.

## Method

### Participants

The sample included PTPP FOs, all of whom had previously completed the PTPP’s training on effective genetic counselling. The training materials for the FOs on how to use the DeSIRe were developed to build on this previous training. FOs were recruited by email via the PTPP. Nine FOs volunteered to (i) attend training on how to use the DeSIRe; (ii) subsequently use the DeSIRe with families of individuals with β-TM in routine practice, and (iii) participate in an interview study. The FOs were from seven cities of the Punjab province, including Lahore, Sheikhupura, Nankana Sahab, Kasur, Gujranwala, Multan, and Faisalabad. These FOs received training in December 2020 and then refresher training in March 2021. Details of this training can be found elsewhere [10]. They then used the DeSIRe in at least three family meetings during April to June 2021. These FOs were subsequently contacted by a researcher by telephone to gain consent and arrange an interview. All nine FOs participated in the interview study.

### Procedure

The interview guide was developed to explore FOs’ experiences of using the DeSIRe in the family meetings, using the Normalisation Process Theory (NPT) as the framework for exploring their perceptions of the potential implementation and sustainability of the DeSIRe. Semi structured interviews were carried out by HJ in Urdu, in person with the two FOs based in Lahore and by telephone with the other FOs based in the other cities. The interviews lasted approximately 40 min, were audio-recorded, translated, and transcribed in English by a bilingual researcher (WNA). SA assured the quality of the interviews, translation and transcription by listening to audio-recorded interviews, reading the transcripts, and conducting the analysis simultaneously.

### Analysis

Reflexive thematic analysis was used to guide data analysis [11, 12], underpinned by the NPT as the theoretical lens for exploring potential implementation and sustainability of the intervention [13]. The analysis involved six phases (see Table 1 for details), using both deductive and inductive approaches [11]. As the most experienced qualitative researcher with expertise on the research topic, SA (of Pakistani origin based in the UK) analysed the data [14]. Analysis initially involved classifying and organising data using subheadings based on the interview guide (deductive analysis using N-Vivo 12, Sage Publications). SA further reviewed the data, modifying, merging and changing themes iteratively (inductive analysis). Subsequently, researchers based in Pakistan (HJ, WNA and MF) reviewed and commented on the themes to ensure inclusion of any cultural nuances from their perspective, which was important because this was an international collaborative study.

**Table 1:** Process of coding and thematic analysis [11].

Phase 1 Familiarisation with data	SA listened to the interview audio recordings in Urdu, read and re-read the transcripts in English. HJ conducted the interviews, and WNA transcribed the data.
Phase 2 Generating initial codes	SA initially generated codes (using NVivo 12) based on questions in the interview guide, then identified patterns of meaning beyond the scope of the interview guide.
Phase 3 Searching for themes	As a starting point, the initial codes were categorised according to the topics in the interview guide (deductive analysis).
Phase 4 Reviewing potential themes	SA reviewed, added, modified, merged and changed these initial themes as analysis progressed (inductive analysis), to better understand FOs’ experiences and perception of implementing and sustaining the DeSIRe in the PTPP.
Phase 5 Defining and naming themes	SA, WNA, MF and HJ discussed, refined and agreed the names and interpretations of the themes. The inclusion of these researchers’ subjectivity during this phase led to a more nuanced understanding of the data in this international collaborative study [17].
Phase 6* Producing the report	SA produced most of the first draft of the manuscript. HJ and MA initially drafted the discussion because of their clinical expertise. All the authors contributed to reviewing and revising the manuscript.

*Analysis involved moving back and forth between the phases.

## Results

Interviews were conducted with nine FOs. All the FOs were male, with a mean age of 33.00 years (ranging from 22 to 45 years). Four of the FOs were educated to intermediate level (equivalent to the UK A level), and five were educated to degree level. The number of years the FOs had worked for the PTPP ranged from 2 to 10.

### Qualitative findings

The qualitative findings are presented below with illustrative quotes from participants, attributed anonymously. To protect their anonymity, the quotes do not indicate the cities in which the FOs were based.

#### Perceived purpose and value of the DeSIRe for FOs

The FOs acknowledged that they understood the purpose of the DeSIRe as enabling them to facilitate informed decision making for cascade screening. They explained that their role previously involved the provision of similar information, with the aim of advising relatives to opt for carrier testing. The FOs understood that the DeSIRe was designed to enable FOs to provide more detailed and tailored information to relatives, in order to support relatives in making screening decisions for themselves:

“…now the right to decide lies with them… We just provide them with comprehensive information and they can decide the rest for themselves.” (FO7)

The FOs talked about the value of the DeSIRe for their own practice. They appreciated how the use of the DeSIRe enabled them to provide information more effectively and efficiently compared to their existing resources. They valued how the DeSIRe enabled them to provide more detailed information in a concise and structured format, using simple and accessible language for individuals with varying information needs and educational backgrounds.

Use of the DeSIRe was believed to save time, because it contained the right level, amount and type of information, addressing relatives’ information needs. Therefore, relatives had comparatively fewer questions, hence less time was needed to answer them. Use of the DeSIRe also made it ‘easier’ for FOs to deliver information to more relatives in a meeting.

“Using this leaflet saves our time, as a lot of families understand most of it just by reading it, so there are very few questions left to answer. Moreover, work pressure is reduced as we can deal with a greater number of people at one time.” (FO1)

“…this has helped us understand how best to guide people. We can systematically explain it from the ‘condition’ through to ‘prevention’.” (FO9)

In addition, the FOs stated that the DeSIRe helped them improve their genetic counselling skills, resulting in a more professional genetic counselling meeting. They appreciated that the use of the DeSIRe resulted in a more interactive counselling session, by enabling them to engage in a two-way conversation with relatives. They explained that the DeSIRe empowered and encourage relatives to engage in the thought process required for decision-making about cascade screening, where they felt at ease to ask appropriate questions and clarify information:

“…In the past, it was like a lecture… one-way communication. But this leaflet encouraged them to brainstorm and come up with different questions.” (FO8)

Moreover, the FOs explained that in their previous practice relatives would usually refuse cascade screening because of their poor understanding of its relevance. The FOs believed that using the DeSIRe enabled them to inform relatives more effectively than previously, with the result that relatives, including those with little or no education, gained a better understanding of the relevance of cascade screening. This improved understanding was believed to be the reason for an increased number of relatives making informed decisions about cascade screening:

“This leaflet answers all their (relatives’) questions. They said that previously they failed to get satisfactory answers, so they couldn’t reach a decision.” (FO2)

“…we used to call them and now they call us for carrier testing, solely because it (DeSIRe) has improved their understanding.” (FO4)

“…even less educated people can understand it well.” (FO6)

FOs also valued the DeSIRe because relatives’ improved understanding of cascade screening enabled FOs to achieve their screening targets without coercing relatives. FOs explained how they used to be anxious about meeting performance targets. However, the DeSIRe now enabled them to concentrate on a more supportive role, helping relatives understand the implications of carrier testing, while also resulting in the FOs achieving their screening targets:

“…it was all about achieving our target… now we are relaxed and focus more on better explaining the disease, as they are the ones to decide.” (FO7)

“…they (relatives) used to ignore us. But with this (DeSIRe), we have seen more interest, and people approach us wanting to learn their carrier status.” (FO9)

Overall, the FOs believed that the DeSIRe was an important intervention to support genetic counselling about cascade screening. They were keen to change their current practice of ‘advising’ relatives to using the DeSIRe to ‘support’ relatives to make decisions for themselves. By using the DeSIRe, they felt that they were more supportive in facilitating informed decision making rather than advising or making decision for the relatives:

“…it’s their right to decide if they want to have testing or not. Our goal is to explain in detail so that they understand the need for testing.” (FO3)

#### Perceived value of the DeSIRe for relatives

The FOs also talked about why the use of the DeSIRe was in the best interest of relatives. They said it included all the appropriate information required by relatives to support their decision-making. In particular, they experienced how relatives appreciated the ‘value clarification exercise’. FOs believed it enabled relatives to clarify their own values, consider what was important or mattered most to them, and hence identify their own preferences:

“One of the best things in this leaflet is the questionnaire… It helps relatives to think and decide for themselves if they should or shouldn’t get tested.” (FO1)

Based on their observations, the FOs believed it was important to provide relatives with the DeSIRe for them to refer back to in their own time, and that relatives also valued the DeSIRe:

“ …they can revisit it and refresh their knowledge.” (FO4)

“…if families misplace their leaflet, they contact us for another copy because they find it so useful.” (FO9)

#### Perceptions of implementing and sustaining the DeSIRe

The FOs were highly supportive of implementing the DeSIRe more widely in the PTPP. They believed that the appropriateness of information presented in the DeSIRe would equip other FOs to better perform their role. They also felt that it would be more cost effective if a similar approach using the DeSIRe was implemented in other areas. They also suggested that it should be adopted by organisations in the other provinces of Pakistan:

“It should be used by all FOs in all districts (in the Punjab).” (FO6)

“It (DeSIRe) will help increase FOs’ knowledge, broaden their mind… make their job of counselling even easier… help them provide information… more efficiently.” (FO1)

FOs believed the DeSIRe could also be a useful resource for other healthcare professionals who had contact with children with β-TM or their parents, including genetic counsellors, paediatricians and family physicians:

“…anyone can use it, as the language used is very simple and easy to understand. Any health worker… genetic counsellor, lab technician…” (FO7)

“…it can be used in chelation centres… thalassemia treatment centres” (FO5)

FOs showed their satisfaction with the training they were provided. To implement the DeSIRe more widely, they acknowledged the need for training for other colleagues and mutual support for co-operative working. They agreed that the training they received was sufficient and could be replicated for other colleagues. Furthermore, some FOs also showed their willingness to advocate the DeSIRe:

“…the training made me confident of improving my work to provide the information using this leaflet.” (FO8)

“It would be helpful if we arranged a meeting with other FOs, where we comprehensively discuss how and why to use it.” (FO1)

Furthermore, the FOs acknowledged the need for policy making and support for implementation at a higher level. They suggested the Department of Health should support the use of the DeSIRe in routine practice through the provision of resources, and should also raise awareness about β-TM more widely using the DeSIRe:

“Yes, we really need the government’s support to raise awareness of this disease through the media… all the information in the leaflet.” (FO8)

The FO did not raise any concerns about the use of the DeSIRe in their clinical practice, or any barriers or challenges to implementing the DeSIRe.

## Discussion

The FOs valued the use of the DeSIRe in clinical practice to enable them to facilitate decision-making about cascade screening. In line with requirements for implementing such interventions [15, 16], our study shows that the FOs had positive attitudes toward using the DeSIRe in their routine clinical practice, changing their role from advising to facilitating relatives’ decision-making, and adapting their communicative skills. Overall, this study provides valuable insight into why and how the DeSIRe could be implemented and optimised in clinical practice for cascade screening in Pakistan.

The FOs’ positive attitudes towards using the DeSIRe were based on their comparisons of this intervention with current PTPP resources, practice, and emphasis on screening uptake targets. Similar to other studies [7, 17], the FOs agreed with the clarity and level of information for relatives and believed the DeSIRe was helpful for enhancing relatives’ awareness of β-TM and personal risk of being a thalassaemia carrier. The FOs recognised that well-informed relatives essentially made their work easier, saved time, and helped them reach their screening targets [7]. Overall, FOs believed the use of the DeSIRe was more efficient and effective for improving uptake of cascade screening. Following discussions based on these findings with the Director of the PTPP, our understanding is that the DeSIRe will be implemented within the PTPP, and that screening targets (set by the Director in consultation with the organisation’s advisory committee) will remain the same. Further research is needed to estimate the expected cost-effectiveness of using the DeSIRe compared to current resources on the uptake rate of cascade screening and subsequent identification of thalassaemia carrier, including the costs incurred by the health system.

Moreover, the FOs’ believed the DeSIRe had a positive impact on their own knowledge, their genetic counselling approach, and performance. They recognised the DeSIRe enabled them to play a more supportive role by engaging relatives in SDM rather than being directive, and associated this with relatives’ increased interest in cascade screening. These findings may explain why FOs were willing to adapt their communication skills by using the DeSIRe and shift from using a directive approach to engage relatives in SDM. Unlike Western countries, there is little emphasis in healthcare policies in Pakistan on the need for non-directiveness, SDM or ‘patient-centered’ healthcare. Lack of such policies are an indication of a tension between the population goals of clinical genetic services (reducing the birth prevalence of β-TM by persuading individuals) and the goals of supporting individual patients and their relatives to make autonomous decisions [18, 19]. Our findings suggest that implementation of the DeSIRe would support both goals, i.e. enable FOs to reach their screening targets by supporting individual decision-making. Therefore, the PTPP should develop policies that recognise and promote individual choice and emphasise the importance of SDM. Such policies could lead to an organisational culture shift from a focus on screening targets (population goals using coercion) to supporting relatives to engage in informed decision-making.

Furthermore, genetic counselling is mainly a Western concept and profession, which has been adapted in an ad-hoc way in low-middle income countries [20], often with sparse training and resources. The FOs in this study had already received PTPP training on effective genetic counselling. Nevertheless, our findings suggest that implementation of the DeSIRe and its training resources could further enable a cultural shift to non-directiveness and SDM [21]. The DeSIRe, together with the implementation toolkit, will be made available via a website for adoption and use more widely in Pakistan, and in other populations. The DeSIRe training materials for healthcare professionals were developed to build on their previous training on genetic counselling. Therefore, adoption of the DeSIRe training would require consideration of healthcare professionals’ previous training in genetic counselling.

FOs were also supportive of implementing the DeSIRe more widely. However, the challenge to advocating for the wider implementation of the DeSIRe in Pakistan is the lack of organisations similar to the PTPP in the other four provinces, where cascade screening is offered on a more ad-hoc basis mainly via non-government organisations (NGOs) for thalassaemia. Therefore, further research is needed on the reach, acceptability, and implementation of the DeSIRe via NGOs. In parallel, our research can inform the dialogue required with policy makers to develop and implement government funded services, similar to the PTPP, more widely in Pakistan. Implementation of a national prevention programme, including the use of the DeSIRe, could lead to increased awareness and uptake of cascade screening to manage the burden of β-TM in Pakistan.

FOs also valued the DeSIRe because they observed how it was appreciated by relatives. Similar to our other research, FOs believed the DeSIRe was beneficial for relatives, both during the meeting and afterwards to use as a resource for their own reference and also, if preferred, to share this more widely with other relatives [10]. Additionally, the FOs recognised the DeSIRe was designed for use with people with varying degrees of comprehension and were confident that individuals with low or no literacy will also benefit from it.

Since this study was conducted, the remit of the PTPP has been expanded to include other genetic conditions, and it is now recognised as the Punjab Thalassaemia and other Genetic Disorders Prevention and Research Institute (PTGD). Based on the perceived positive value of the DeSIRe by FOs in this study, and by relatives [10], the DeSIRe has the potential for further patient benefit through being adopted, with adaptations as appropriate, in the PTGD, for other genetic conditions where similar decisions will be required.

### Strengths and limitations

This is the first study to explore healthcare professionals’ perceptions of implementing a decision support intervention for cascade screening for β-TM, adding to the scarce literature in this field in low-middle income countries. Our findings are not intended to be numerically representative. Instead, they present an in-depth insight needed into FOs’ perceptions of the enablers and barriers to implementing the DeSIRe. Our qualitative data has been collected rigorously and substantially represents the breadth of FOs’ experiences of the DeSIRe, providing insight into how the DeSIRe could be used to support informed and shared decision-making [22]. Furthermore, this study was conducted with healthcare professionals who best understand the context and population for which the implementation of the DeSIRe is intended.

The study is limited to the Punjab province. Further research is needed with healthcare professionals, working in both governmental and non-governmental organisation, in the other provinces to explore its potential implementation beyond the PTPP. In addition, the study includes only male participants as the PTPP only has male FOs. Further research on the implementation of the DeSIRe should include the perspectives of other key stakeholders, including female healthcare professionals.

## Conclusion

The FOs valued the use of the DeSIRe to enable them to facilitate decision making about cascade screening, and would support wider implementation of this intervention. A large-scale RCT is now needed to evaluate the effectiveness of the DeSIRe for improving informed decision-making about cascade screening.

## Data Availability

The datasets generated and/or analysed during the current study are available from the corresponding author on reasonable request.
